# Clinicopathological Behavior and Treatment-related Outcome of Rare Salivary Duct Carcinoma: The Shaukat Khanum Memorial Cancer Hospital Experience

**DOI:** 10.7759/cureus.3139

**Published:** 2018-08-14

**Authors:** Abdul Wahid Anwer, Muhammad Faisal, Mohammad Adeel, Omer Waqas, Muhammad Abu Bakar, Saman Qadeer, Maliha Koukab, Raza Hussain, Arif Jamshed

**Affiliations:** 1 Department of Surgical Oncology, Shaukat Khanum Memorial Cancer Hospital and Research Center, Lahore, Pakistan, Lahore, PAK; 2 Department of Surgical Oncology, Shaukat Khanum Memorial Cancer Hospital and Research Center, Lahore, PAK; 3 Department of Surgical Oncology, Shaukat Khanum Memorial Cancer Hospital and Research Center, Lahore, Pakistan; 4 Pathology, Shaukat Khanum Memorial Cancer Hospital and Research Center, Lahore, PAK; 5 Biostatistician and Cancer Epidemiologist, Shaukat Khanum Memorial Cancer Hospital and Research Center, Lahore, PAK; 6 Surgical Oncology, Shaukat Khanum Memorial Cancer Hospital and Research Center, Lahore, PAK; 7 Department of Radiation Oncology, Shaukat Khanum Memorial Cancer Hospital and Research Center, Lahore, PAK

**Keywords:** salivary duct carcinoma, salivary gland tumors, head and neck surgery

## Abstract

Background

Salivary gland tumors are rare salivary gland malignancies with resemblance to ductal breast carcinoma. We have described clinicopathological behavior and treatment outcomes of this rare malignancy.

Methods

Salivary duct carcinoma patients treated from 2010 to 2015 were retrospectively analyzed for clinicopathological characteristics and treatment-related outcomes of the disease.

Results

A total of 12 patients with salivary duct carcinoma were included in the study. All were males with mean age of 52.58 ± 13.43. Parotid gland was the most commonly involved major salivary gland while buccal mucosa and anterior tongue were most common oral cavity sub-sites involving minor salivary glands. The disease-free survival was 75% at 10 months and 25% at 20 months. The mean follow-up time was 12 months. There were three local recurrences and one distant metastasis.

Conclusion

Salivary duct carcinoma is a locally aggressive tumor with tendency for local recurrence and distant metastasis. Adverse features such as perineural invasion, extra-capsular spread and advanced nodal disease may worsen prognosis.

## Introduction

Salivary duct carcinomas are rare tumors representing 10% of salivary gland malignancies. They may arise de novo or secondarily in a pre-existing pleomorphic adenoma. The histological features resemble to invasive ductal carcinoma of the breast and require immunohistochemistry to rule out metastasis among patients with previous history of breast carcinoma [1]. Salivary duct carcinomas have been classified as high-grade malignancies [2]. The standard treatment for salivary duct carcinoma of parotid gland is total parotidectomy, ipsilateral neck dissection followed by postoperative radiation therapy with or without concurrent chemotherapy; however, salivary duct carcinoma of parotid gland has grave dismal prognosis and chemotherapy may have a palliative role in metastatic disease [3].

Salivary duct carcinoma is a rare tumor, so limited studies have been published. We aim this study in describing clinicopathological behavior and treatment-related outcomes such as disease-free survival, patterns of failure and adverse pathological features affecting survival at a high volume tertiary care cancer center.

## Materials and methods

All patients’ record was retrieved from the Cancer Registry Database of Shaukat Khanum Memorial Cancer Hospital. The patients with histological diagnosis of salivary duct carcinoma were selected from the database. Demographic records for each individual including age at diagnosis, gender, grade, stage, geographic location, treatment modality and follow-up were all retrospectively analyzed from the same database. All patients had a baseline computed tomography (CT) scan or magnetic resonance imaging (MRI) of the primary site. The study was exempted by the Institutional Review Board (IRB). Data were analyzed using IBM SPSS Statistics for Windows, Version 20.0 (released 2011, IBM Corp., Armonk, NY). Kaplan–Meier curves were used to analyze survival data.

## Results

**Figure 1:**
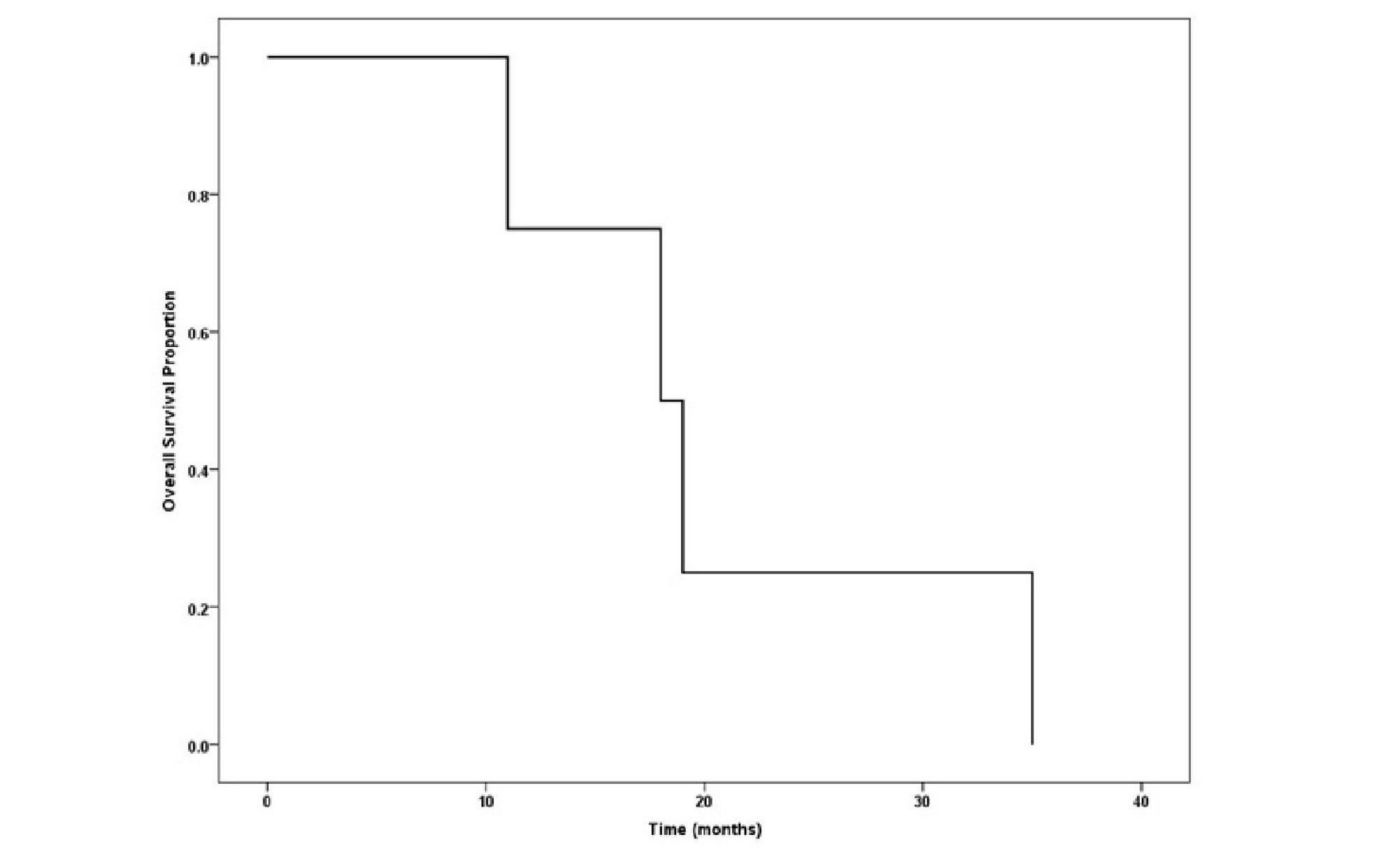
Disease-free survival (DFS) in months.

A total of 12 patients' records, diagnosed with salivary duct carcinoma of head and neck region from 2010 to 2015, treated at Shaukat Khanum Memorial Cancer Hospital, were retrospectively analyzed to describe the clinicopathological characteristics and treatment-related outcomes of the disease (Tables 1, 2). All patients were males with the mean age of 52 years at presentation (Range 29–71). Primary site of involvement was parotid (n = 7), submandibular gland (n = 3), buccal mucosa (n = 1) and anterior tongue (n = 1). There was only one patient who was treated with palliative intent while remaining 11 underwent surgery followed by adjuvant treatment. Ipsilateral neck dissection was performed in five patients as nodal disease was evident on imaging studies. Majority of patients had R1 (microscopic residual disease) and R2 (macroscopic residual disease) resections (n = 8) with only two patients having clear resection surgical margins. There were four patients with positive perineural invasion and four with lympho-vascular involvement. Mean follow-up time was 12 months (Range 4–30). The Kaplan–Meier survival curve showed dismal results at 20 months after treatment (Figure 1). Mean dose for post-operative radiotherapy was 50.5 Grey given as daily 2 Grey/fraction five days/week over five weeks.

**Table 1:** Clinical and demographic features. "x" denotes disease which cannot be assessed.

Variables		Characteristics	Frequency N (%)
Age in years		Mean ± SD*	52.58 ± 13.43
Ethnicity		Afghanistan	2 (16.7%)
		Gilgit-Baltistan	1 (8.3%)
		Khyber Pakhtunkhwa	3 (25.0%)
		Punjab	6 (50.0%)
Site		Oral cavity	2 (16.7%)
		Salivary glands	10 (83.3%)
Subsite		Buccal mucosa	1 (8.3%)
		Parotid	7 (58.3%)
		Submandibular	3 (25.0%)
		Tongue anterior	1 (8.3%)
Clinical stage		X	1 (8.3%)
		1	2 (16.7%)
		2	1 (8.3%)
		3	1 (8.3%)
		4	7 (58.3%)
Pathological stage		X	2 (16.7%)
		1	3 (25.0%)
		2	3 (25.0%)
		3	1 (8.3%)
		4	3 (25.0%)

**Table 2:** Clinicopathological characteristics. RT: Radiotherapy; PNI: Perineural Invasion; LVI: Lymphovascular Invasion; ECS: Extracapsular Spread; M: Male; p: Pathological; NA: Not Applicable.

Serial No	Age/ Gender	Site	Treatment	Pathological stage	Adjuvant	Recurrence	Follow up (months)	Status
1	51/M	Parotid	RT	NA		No	10	Alive
2	52/M	Tongue anterior	Surgery	pT2N0 Re-excision No PNI/LVI	NA	Regional	13	Alive
3	45/M	Buccal mucosa	Surgery	pT1N0 PNI+, Close Margin	RT	No	10	Alive
4	29/M	Parotid	Surgery	pT2N0 close margin	RT	No	9	Alive
5	64/M	Submandibular	Surgery	pT3Nx PNI/LVI + Close margin	RT	No	4	Died
6	66/M	Submandibular	Surgery outside	T4bN3	RT	No	6	Died
7	66/M	Submandibular	Surgery	T2Nx	NA	NA	4	Lost to Follow up
8	42/M	Parotid	Surgery	pT3N0	RT	Distant	30	Alive
9	63/M	Parotid	Surgery	pT4aN3b PNI + ECS+ Close margin	RT	Local (Intracranial Extension)	30	Alive
10	36/M	Parotid	Surgery	pT3N3 PNI/LVI + ECS+ Close margin	RT	Local	22	Alive
11	46/M	Parotid	Surgery	pT1Nx PNI+, Involved margin	RT	No	20	Alive
12	71/M	Parotid	Surgery	pT1Nx	RT	No	5	Alive

## Discussion

**Figure 2:**
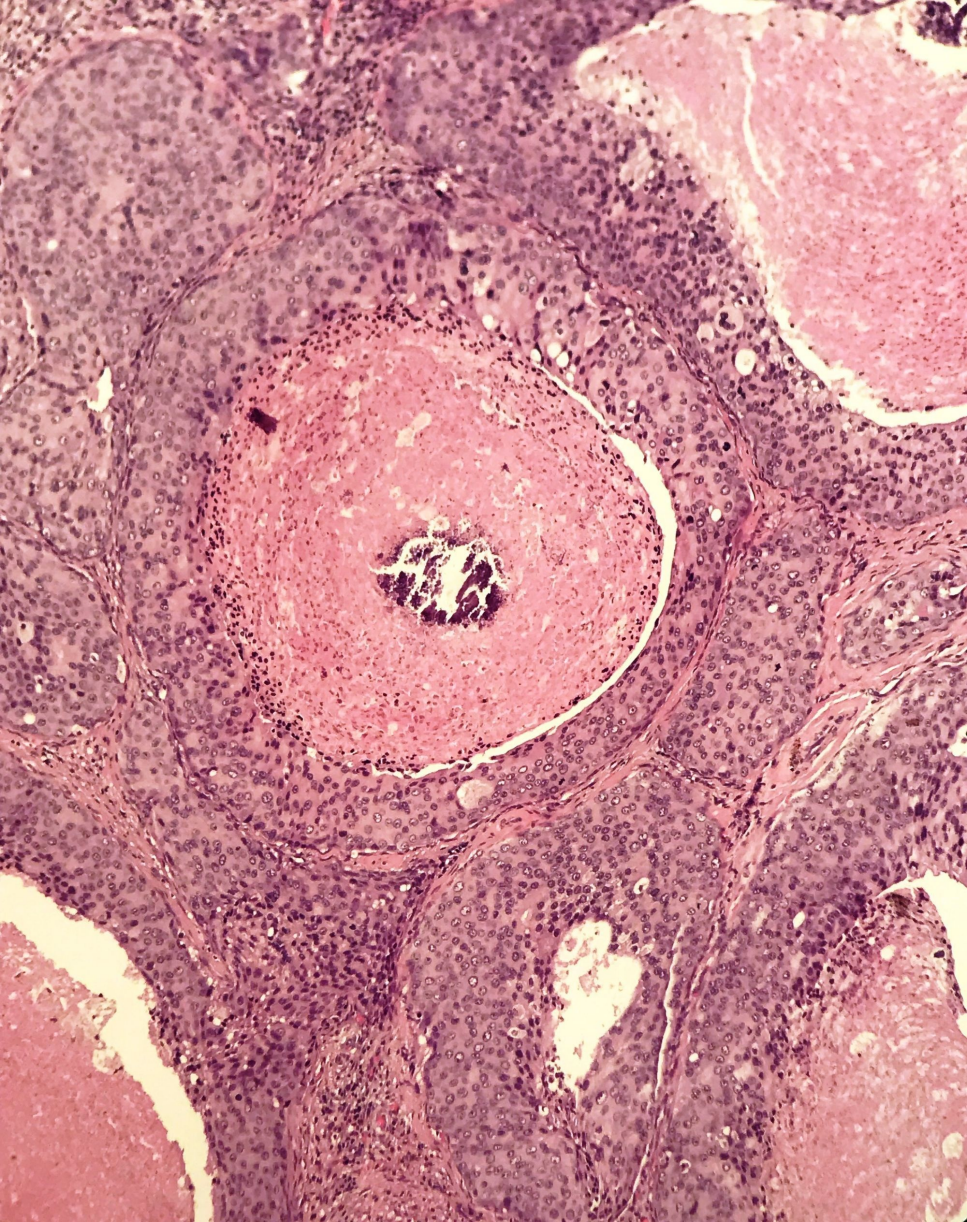
Lobules displaying central comedo-like necrosis and microcalcifications, similar to the ductal carcinoma in situ (DCIS) of the breast. (H&E, 20x Magnification)

**Figure 3:**
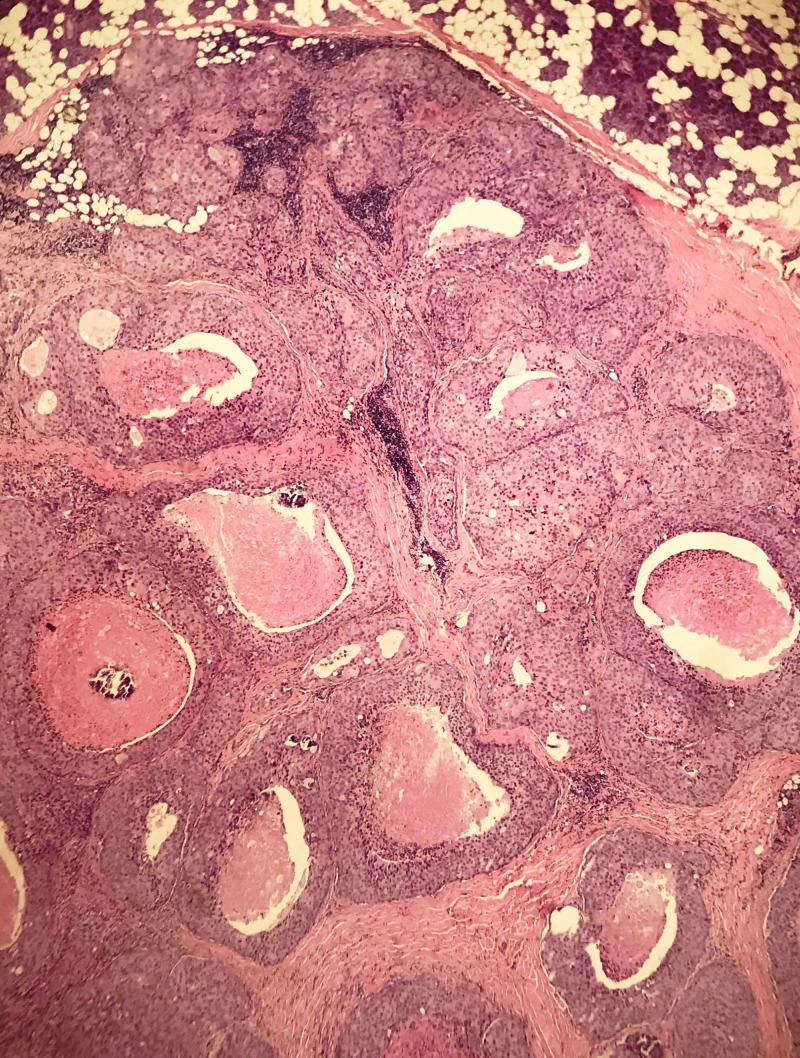
Salivary duct carcinoma, displaying prominent eosinophilic appearance, rounded and variably sized solid and cystic lobules. (H&E, 10x Magnification)

**Figure 4:**
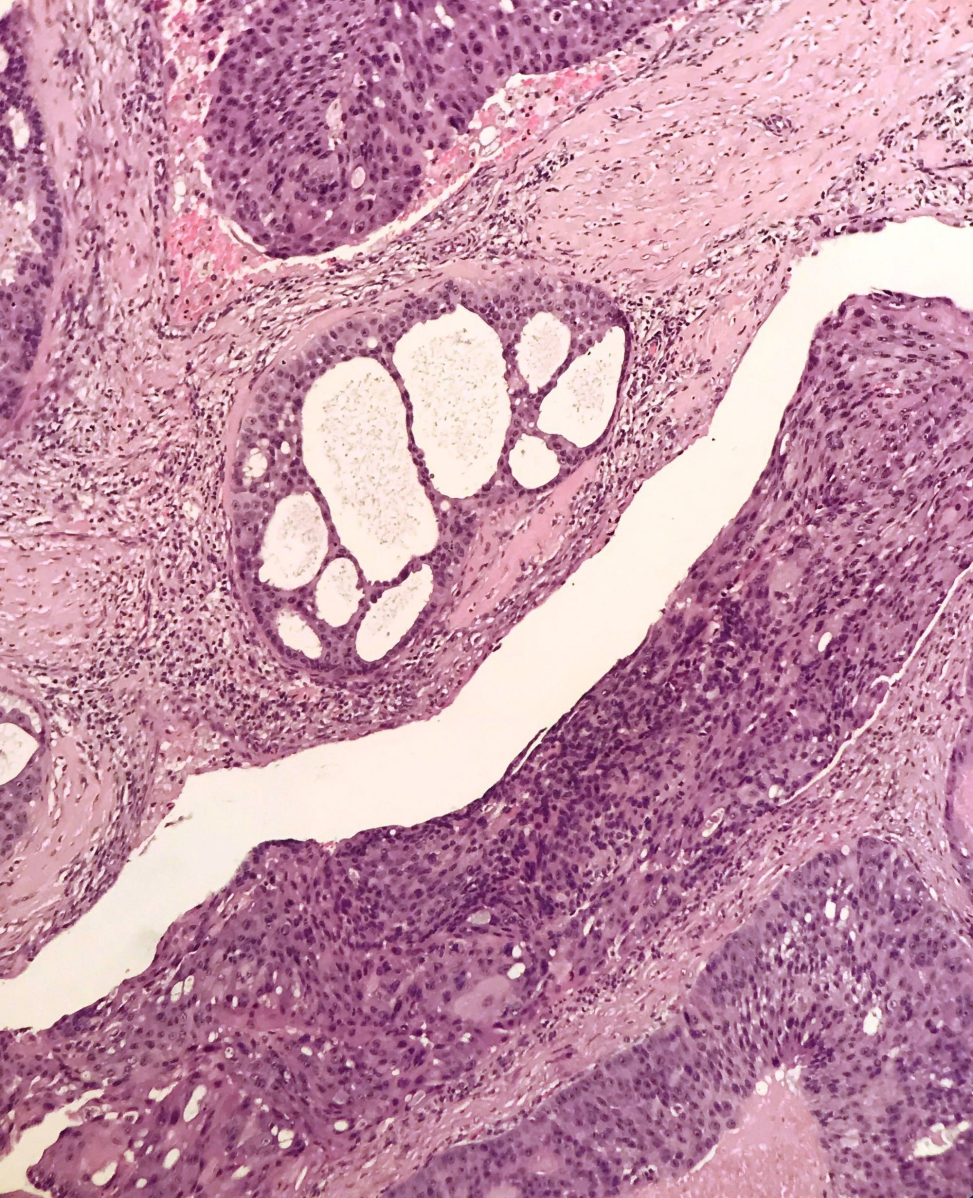
A focus showing cribriform nest (H&E,10x Magnification)

**Figure 5:**
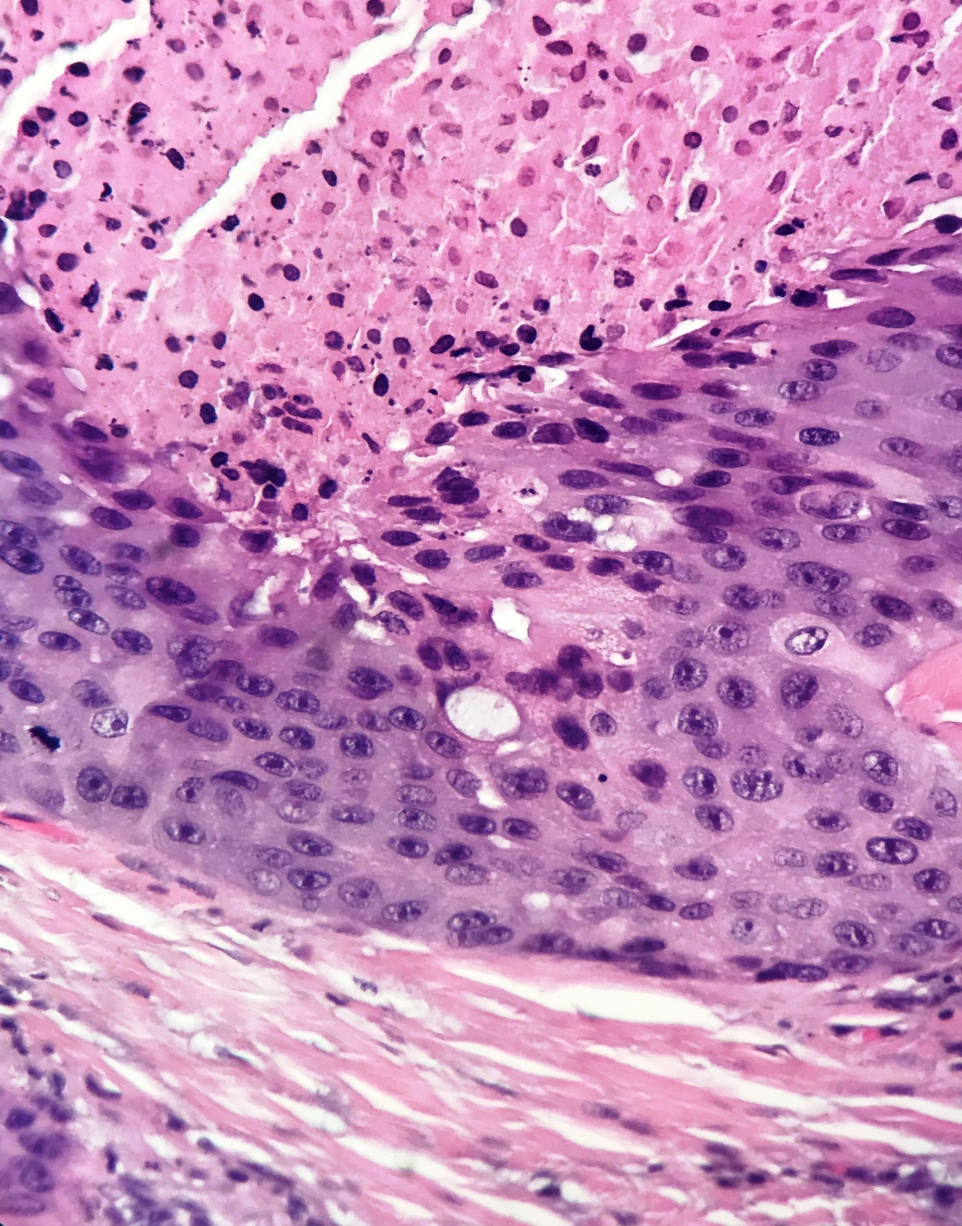
Tumor cells showing abundant eosinophilic cytoplasm, hyperchromatic nuclei with visible nucleoli. (H&E, 40x Magnification)

**Figure 6:**
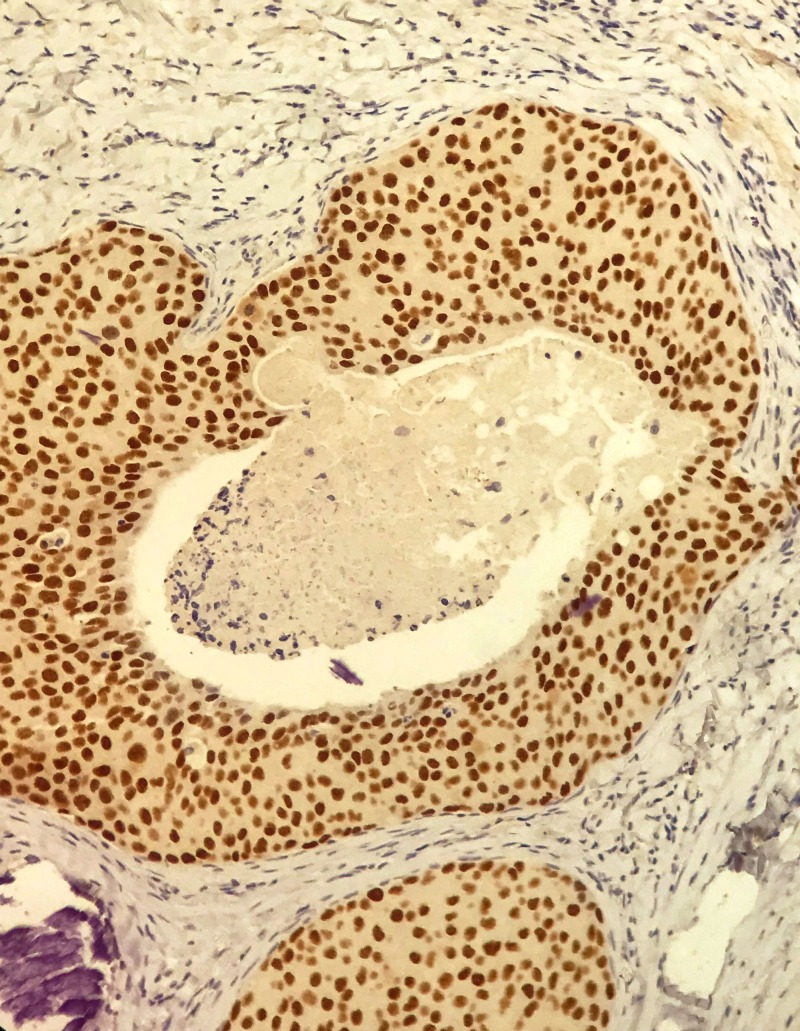
Nuclear positivity of androgen receptor (AR) among the tumor cells.

Kleinsasser et al. have used the term ‘salivary duct carcinoma’ due to its resemblance to ductal carcinomas of the breast. Reported case series have re-classified many of these tumors into epithelial-myoepithelial and polymorphous low-grade adenocarcinoma resulting in difficulty of establishing the actual incidence [4]. The majority of case series and reports have shown aggressive nature of the disease, propensity for nodal metastasis and strong tendency for loco-regional recurrence and distant metastasis. The close resemblance to ductal carcinoma of the breast is attributed to comedo-type necrosis and calcifications microscopically and cystic changes macroscopically [5] as shown in Figures 2, 3. The poor prognosis and aggressive nature of these tumors have suggested that accurate diagnosis so that timely intervention may improve outcomes. Cytological literature comprises more of case reports and small case series [6,7]. Cribriform, trabecular, acinar and papillary formations can be seen (Figure 4). The individual cells are large, monomorphic to pleomorphic, and polygonal to cuboidal, with abundant, finely granular cytoplasm (Figure 5). Squamoid and oncocyte-like appearances are also seen [8]. Due to similar nature of salivary duct carcinoma and breast ductal carcinoma, over-expression of androgen receptor, human epidermal growth factor receptor 2 (HER-2)/neu proto-oncogene has also been studied (Figure 6). The reported rate is around 20–25% and this trend is similar to our case series where two of our patients were human epidermal growth factor receptor (HER-2) positive while one was androgen receptor positive (Figure 5) [9,10]. The differential diagnosis for high-grade salivary duct carcinoma includes the papillary cystic and microcystic variants of acinic cell carcinoma, metastatic squamous cell carcinoma, metastatic breast cancer, melanoma, mucoepidermoid carcinoma, and oncocytic carcinoma. Based on nuclear findings, it may be possible to distinguish salivary duct carcinoma from other high-grade salivary gland carcinomas, by immunostaining for androgen receptor, gross cystic disease fluid protein-15 and p63 [11,12].

Parotid gland is the more commonly involved site (88%) in literature followed by submandibular gland (8%). Men are three times more likely to get salivary duct carcinoma than women [13,14]. Rarely, there is involvement of minor salivary glands and larynx [15-17]. Among salivary glands, parotid gland involvement was found to be in majority (70%) of cases followed by submandibular gland (30%). Most commonly involved oral cavity sub-sites were buccal mucosa and anterior tongue.

Due to high incidence of loco-regional recurrence and distant metastasis resulting in dismal survival outcome, aggressive approach should be the mainstay of treatment. Local failure was observed in three of our patients while one developed distant metastasis. Delgado et al. have suggested preserving facial nerve if not involved while Colmenero Ruiz et al. have advocated sacrificing the nerve even in superficial tumors [18,19]. The reported incidence of neck metastasis is 65% thus elective neck management should be recommended. Other adverse features such as perineural/lympho-vascular invasion may have a role in local recurrence other than close or positive margin. Only 25% of our patients developed local recurrence and both were positive for perineural invasion and extra-nodal extension. Only 20% of patients had clear surgical margins while 40% had close and 40% had involved margins. None of the patients with involved margins developed local recurrence. Adjuvant radiotherapy has shown to be effective in terms of survival outcome in literature but small number of patients in reported series and retrospective studies have raised a concern whether or not it should be a part of treatment guidelines [13]. Adjuvant radiotherapy was given to nine of our patients and only two have developed local recurrence while one had distant failure. In breast cancer, detection of human epidermal growth receptor 2 (HER-2) gene amplification increases the identification of responders to targeted therapy [20-22]. Metastatic salivary duct carcinomas have shown good response to trastuzumab [23-25]. This may open up further avenues in determining the role of adjuvant chemotherapy for immunohistochemical marker positive tumors. Previous studies have shown that advanced nodal disease, lymphovascular invasion and extra-capsular spread have negative impact on survival [26]. Estimated disease-free survival in our series stayed at 75% at 10 months but control rates were poor (25%) at 20 months. The cases with local recurrence were both positive for perineural invasion, extras-capsular spread and having an advanced nodal disease. The limitations of this study are retrospective nature, small sample size and short follow-up.

## Conclusions

Salivary duct carcinoma is among the high grade aggressive salivary gland tumors resembling ductal carcinoma of the breast. Early recurrence and strong tendency for local and distant metastasis have worse impact on survival. Surgery followed by radiotherapy to both primary and cervical bed seems to be the preferred treatment. Due to increased likelihood of cervical metastasis, elective neck management should be performed in these tumors.
